# New National Health Interview Survey Occupational Health Supplement Topic Page

**Published:** 2013-07-19

CDC’s National Institute for Occupational Safety and Health (NIOSH) has released a new topic page at http://www.cdc.gov/niosh/topics/nhis. The page describes the 2010 National Health Interview Survey (NHIS) Occupational Health Supplement (OHS), and results are organized into National Occupational Research Agenda (NORA) sector profiles. These profiles each contain charts and tables describing the prevalence rates of certain work-related health conditions, work organization factors, and occupational psychosocial and physical/chemical exposures. Results are categorized by industry and/or occupation subgroups within each sector profile. An additional profile compares sector-level results and includes some additional outcomes that cannot be shown for subsectors because of small subsamples.

Using the information available on this page, employers can benchmark the health of their workers against industry averages. Additionally, industry and employer stakeholders can use the information to prioritize interventions at the industry and organizational levels. This helps ensure that the most pressing industry health concerns receive appropriate attention.

In addition, NIOSH is looking for input on what questions to include in the 2015 NHIS-OHS. Additional information is available at http://blogs.cdc.gov/niosh-science-blog/2013/06/24/nhis.

